# Deep Learning for Generating Time-of-Flight Camera Artifacts

**DOI:** 10.3390/jimaging10100246

**Published:** 2024-10-08

**Authors:** Tobias Müller, Tobias Schmähling, Stefan Elser, Jörg Eberhardt

**Affiliations:** 1 Institute for Photonic Systems Hochschule Ravensburg-Weingarten, University of Applied Sciences, Doggenriedstraße, 88250 Weingarten, Germany; tobias.schmaehling@rwu.de (T.S.); joerg.eberhardt@rwu.de (J.E.); 2 Institute for Artificial Intelligence Hochschule Ravensburg-Weingarten, University of Applied Sciences, Doggenriedstraße, 88250 Weingarten, Germany; stefan.elser@rwu.de

**Keywords:** time-of-flight, learning-based simulation, domain transfer

## Abstract

Time-of-Flight (ToF) cameras are subject to high levels of noise and errors due to Multi-Path-Interference (MPI). To correct these errors, algorithms and neuronal networks require training data. However, the limited availability of real data has led to the use of physically simulated data, which often involves simplifications and computational constraints. The simulation of such sensors is an essential building block for hardware design and application development. Therefore, the simulation data must capture the major sensor characteristics. This work presents a learning-based approach that leverages high-quality laser scan data to generate realistic ToF camera data. The proposed method employs MCW-Net (Multi-Level Connection and Wide Regional Non-Local Block Network) for domain transfer, transforming laser scan data into the ToF camera domain. Different training variations are explored using a real-world dataset. Additionally, a noise model is introduced to compensate for the lack of noise in the initial step. The effectiveness of the method is evaluated on reference scenes to quantitatively compare to physically simulated data.

## 1. Introduction

Time-of-Flight cameras have gained increasing attention in recent years for their ability to capture 3D scenes in real-time. By employing a low-cost sensor coupled with an active light source, ToF cameras measure the distance to objects based on the time it takes for light to travel. This technology offers distinct advantages for machine vision applications, as it requires minimal processing power while delivering reliable data.

The compact design of ToF cameras further expands their range of applications. Smartphones, for instance, can benefit from ToF cameras in face authentication and mobile 3D scanning. In the automotive industry, ToF cameras enable gesture control systems that remain unaffected by varying lighting conditions. Moreover, ToF cameras find utility in simultaneous localization and mapping (SLAM) for autonomous vehicles and reality-altering devices. Despite the numerous applications, ToF cameras are still susceptible to various artifacts that compromise the quality of the captured data. To address this issue, algorithms are used to correct the errors [1,2]. However, traditional approaches are limited in their effectiveness to detect and process features.

Consequently, deep learning has emerged as a powerful tool, enabling the extraction of patterns and features from complex data. As a result, many learning-based approaches that aim to remove errors from ToF data have been proposed recently [3,4,5,6]. They either rely on physically simulated synthetic ToF data or employ unsupervised learning methods. However, the quality and content of the training data play a crucial role in the effectiveness of these approaches. While the use of physically simulated synthetic data has shown promising results, it may neglect certain critical real-world components [7]. As neural networks are increasingly used to correct ToF errors, the need for realistic training data continues to grow. To meet this demand, we use the many available datasets [8,9,10] that offer high-quality point clouds but lack ToF camera data. In addition, they do not include the albedo information required for traditional physical simulations. Our learning-based simulation aims to achieve ToF simulation quality by using only point cloud data, without relying on albedo. This simplifies the process and allows us to utilize a much larger set of training data, including real-world scenes with complex geometries. In doing so, we provide a practical approach that leverages widely available data while still ensuring accurate ToF simulation.

This work presents a learning-based approach that uses high-quality laser scan data to generate amplitude modulated continuous-wave (AMCW) ToF camera artifacts. Throughout this paper, we will refer to these simply as ToF camera artifacts. The approach involves domain transfer in two steps. First, the laser scan data are transformed into the ToF camera domain using MCW-Net [11]. The network is trained on RWU3D [12], a real-world dataset consisting of a high-quality laser scan and multiple ToF images. In the second step, a noise model is added to the network’s output to account for the lack of noise in the first step. The noise model is determined experimentally and applied based on the scan data. This will allow researchers to simulate realistic ToF data from high-quality point clouds to generate datasets including both ToF and ground truth. The evaluation of the proposed method is based on the reference scenes presented by Bulczak et al. [13].

## 2. Related Work

Keller et al. [14] describe a simulation framework with all necessary parameters, which is a rough guideline for further work. Peters et al. [15] focus on simulating bistatic effects that occur due to an illumination ring co-positioned to the sensor. Another simulator provided by Keller and Kolb [16] focuses on computing in real time. Their approach includes local illumination with a Lambertian reflection model, to determine high-resolution phase images. Subsequently, to generate flying pixels, they simply downscale the phase images by averaging over small pixel areas. Finally, they add a Gaussian noise model containing a signal-to-noise ratio part and an intensity-related noise to the phase images. Lambers et al. [17] describe a realistic sensor model covering light propagation and illumination as well as physically accurate charge behavior at the readout circuit level of sensor pixels in response to incident photons. Their simulation is limited to scene materials that are Lambertian reflectors and based on the assumption that the modulated light source and the focus point of the camera are located at the same position.

None of the previously mentioned simulators include the modeling of multipath effects, which are significant ToF artifacts. Furthermore, there has been a lack of evaluation regarding the simulated sensor data in comparison to real acquisition data from an actual ToF sensor.

Meister et al. [18] introduced a simulator that tackles the multipath challenge through a global illumination approach. Their method uses bidirectional path tracing, where rays are emitted from both the light source and the camera. By using the local bidirectional reflectance distribution function (BRDF) of the scene surfaces, they compute the second-order ray. To capture multipath effects, the suggested maximum recursion depth is set to 8, which results in a high computational cost. Meister et al. also provide a visual comparison with real data using range images for two test scenes, “corner” and “box”, as well as with a limited quantitative comparison.

In the work of Bulczak et al. [13] a simulation of multipath errors at realistic framerates together with a quantified evaluation for simulated range images is presented. The reflection model, based on BRDF, makes use of measured data from real-world materials. However, the light propagation only accounts for single-bounce multipath, which leads to incorrect simulation for strongly reflecting surfaces. The proposed evaluation is based on three different scene geometries, which are all corners either without a cube, with a cube, or with a shifted cube in the center. Despite the single-bounce limitation, the mean absolute error for two selected materials across all three geometries is approximately 2.5 cm.

Guo et al. [4] created the synthetic dataset FLAT with the help of transient rendering, where the received irradiance of each time frame is simulated. The simulated data are used to train a neural network to mitigate ToF artifacts. There is no evaluation of the simulated ToF data compared to a real sensor, only the training results are evaluated.

Yan et al. [19] concentrate on background irradiance in space and the BRDFs of satellite materials within their simulator. They introduce an improved path-tracing algorithm, which considers the cosine component of the modulated light signal. In the evaluation of one ground test scene, their method performed better than the simulator proposed by Bulzack et al. [13]. Notably, no learning-based methods for simulating ToF data have been identified thus far.

Noise and error removal in ToF sensors has been explored through both physics-based and learning-based approaches.

In physics-based denoising methods, Freedman et al. [1] proposed SRA, a fast and robust solution for removing MPI in ToF sensors using a look-up table-based approach to generate accurate depth estimates from raw ToF measurements. Godbaz et al. [2] provided a comprehensive overview of existing ToF error correction techniques, focusing on modeling MPI and detecting mixed pixels using surface normals. Their method, however, struggles with subtly mixed pixels due to its reliance on surface angle thresholds.

In learning-based denoising approaches, Mutny et al. [3] introduced a machine learning-based correction for MPI using a simulation-driven database with diverse material configurations to address multi-path deviations. Agresti et al. [7] developed a two-stage convolutional neural network that first analyzes the global structure of the scene and then refines details to remove MPI. Guo et al. [4] extended this by tackling dynamic scene artifacts and sensor noise with a two-stage architecture that integrates motion correction and noise attenuation. Su et al. [6] and Marco et al. [20] further enhanced these techniques with encoder–decoder architectures that predict depth directly from raw ToF data, providing real-time performance and robustness to noise and MPI.

More recent work, such as that of Agresti [21] and Buratto [5], applied adversarial learning to further improve denoising and transient image reconstruction in ToF sensors, using both synthetic and real-world datasets.

## 3. Proposed Method

The proposed method aims to perform domain transfer from a highly precise laser scanner depth map to a low-resolution Time-of-Flight camera depth map using a network-based approach. The output of the network is then refined using a noise model to generate a realistic ToF depth map.

### 3.1. Deep Learning for Generating ToF Data

#### 3.1.1. Network Architecture

The network architecture used in this work is a modified version of MCW-Net [11], which is inspired by the encoder–decoder architecture of U-Net [22]. The MCW-Net was originally designed for rain removal, which targets the identification and manipulation of specific image features. The encoder captures the essential features of the image, while the decoder reconstructs the image, applying learned modifications. This structure is equally useful for ToF artifact simulation.

**Figure 1:**
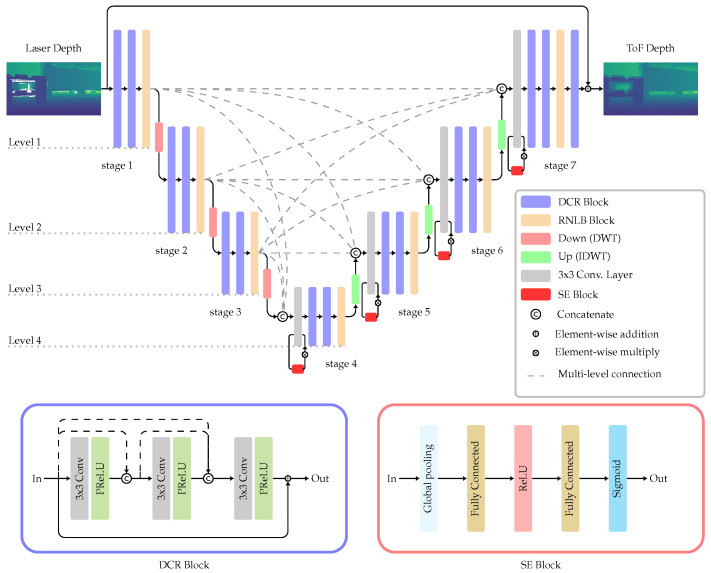
MCW-Net Architecture. The levels represent the size of the feature map. The stages consist of multiple blocks on the same level.

In the encoder phase, the network gradually downsamples the input image to capture lower-resolution representations of the features. These features are then utilized in the decoder phase to generate a new image through an upsampling process. Figure 1 provides an overview of the MCW-Net architecture.

MCW-Net consists of four levels, each representing a specific feature map size. Within each level, a set of layers is defined as a stage. Inspired by U-Net, skip connections are integrated between stages of the same level to mitigate information loss in the second half of the network. MCW-Net additionally connects stages at different levels, as seen in Figure 1. The multi-level connections allow the decoder to access features of varying sizes and levels, which is particularly beneficial when dealing with region-based errors like MPI.

Within each stage of MCW-Net, feature extraction is achieved by combining two densely connected residual (DCR) blocks and a regional non-local block (RNLB). The DCR block consists of three sequential 3 × 3 convolutional layers, followed by a parametric rectified linear unit (PReLU) activation function [23].

The following RNLB further enhances the spatial awareness of the network. It operates by dividing the feature map into patches and performing non-local convolutions. In each non-local operation, the response at a specific position is computed as a weighted sum of features from all spatial positions within the patch. While the original MCW-Net proposed wide rectangular grids for the RNLB [11], a squared grid is implemented in this work. By employing a squared grid, the number of horizontal and vertical features becomes equal, achieving a balanced distribution of information across both dimensions. This allows the network to capture long-range dependencies and contextual information across the feature map.

MCW-Net uses direct wavelet transform (DWT) for downsampling and inverse DWT (IDWT) for upsampling processes. This avoids the information loss caused by conventional subsampling, like max-pooling where only strong features are considered. The DWR is simply implemented by the Haar transform, which consists of four 2 × 2 filters. Therefore, the feature map size is halved and the number of channels quadrupled in the downsampling steps and vice versa in the inverse operation.

The stages in the decoder are designed with a necessary squeeze-and-excitation (SE) block [24] to rescale the feature maps obtained from the multi-level connections; see Figure 1. The number of channels is adjusted by a 1 × 1 convolutional layer.

The scalability of the model is achieved by adjusting the number of channels, as demonstrated in the work of Park et al. [11]. They presented two variants of the model: large and small. The large model contains eight times more channels compared to the small model, resulting in 129.5 million parameters for the large model and 2.2 million parameters for the small model. However, due to the limited amount of training data, experiments revealed that the large model tends to overfit quickly. As a result, subsequent discussions only consider the small version of the model. To properly adapt the network to the depth maps, the input and output channels have been set to one, which is another modification from the original MCW-Net architecture.

#### 3.1.2. Training

**Figure 2:**
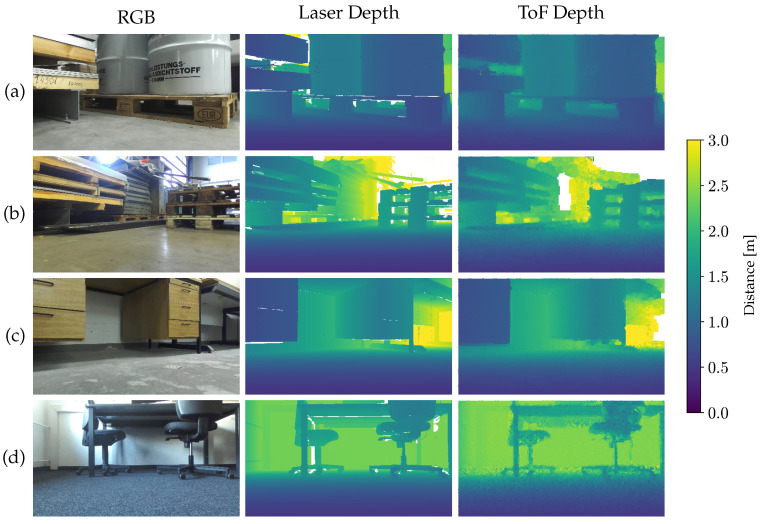
Samples from the Dataset. (**a**,**b**) industrial warehouse, (**c**) basement, and (**d**) office.

The RWU3D dataset is split into 40 train scenes and 17 validation scenes. Figure 2 showcases some sample scenes from RWU3D. Each scene consists of a single laser depth map, which serves as the input image, and a set of *n* ToF depth maps with a size n=20, referred to as labels $Y=\{y_{\left(1\right)},...,y_{\left(n\right)}\}$. During training, a random patch of 256 × 256 is cropped from the input image, and a batch size of 4 is utilized. Not a Number (NaN) values in the input data are appropriately handled by setting them to zero, while NaNs in the labels do not contribute to the loss function. The Adam optimizer is employed, with an initial learning rate of 0.0005 and a learning rate decay of 1/2 every 100 epochs. The model is trained for a total of 400 epochs.

To harness the potential of the dataset, two distinct loss functions were investigated. The first loss function is characterized by the L1 Loss, computed between the mean of all available labels and the network’s prediction $\hat{y}$, as depicted in Equation (1). This approach aims to constrain the network’s prediction to a singular solution instead of considering the 20 different possibilities.

(1) $$L_{\mathrm{mean}}={∥\left(\frac{1}{n}\sum_{k=1}^{n}y_{\left(k\right)}\right)-\hat{y}∥}_{1},$$

On the other hand, the second loss function is determined by calculating the minimum L1 distance among all individual labels in the dataset, as shown in Equation (2). This emphasizes precise predictions, favoring scenarios where multiple outcomes are appreciated.

(2) $$L_{\min}=\min_{k=1,\ldots ,n}{∥y_{\left(k\right)}-\hat{y}∥}_{1}.$$

Data augmentation is applied using horizontal and vertical flipping to increase the diversity of the training dataset. This augmentation is especially important due to the presence of floor areas at the bottom of each scene. Furthermore, the impact of noise on data augmentation is explored. Three distinct input types are employed:

1. No noise
2. Additive Gaussian noise, denoted as [Laser+Noise]
3. Gaussian noise introduced on a separate channel, referred to as [Laser, Noise]

In addition to the enhanced robustness achieved through data augmentation, the implementation also has the objective of ensuring that the network’s output predicts realistic noise characteristics. However, results show that all of the above-mentioned models struggle to produce realistic noise. The model is implemented in Pytorch, and trained on a NVIDIA RTX 3070. The training took approximately 10 h.

### 3.2. ToF Noise Model

The behavior of the utilized model made clear that a noise model is needed. Developing an accurate depth noise model for ToF sensors is still a topic of current calibration research. Thus, similar to [13], a simple noise model is presented. This noise component will be combined with the model’s predictions to simulate realistic noise characteristics.

Various noise sources contribute to the overall noise in ToF cameras. The statistical distribution of ToF cameras can be approximated reasonably well by a Gaussian distribution, as shown in previous studies [16,25]. To quantify the spread of this distribution, three key parameters that affect the noise were examined: distance, angle of incidence, and the presence of edges. These parameters were selected based on the available input data, which is the depth map obtained from the laser scanner. The output functions of the three distinct noise sources are combined to generate a pixel-wise standard deviation.

The standard deviation as a function of distance is approximated by the linear model in Equation (3). The coefficients $k_{0}$ and $k_{1}$ for the linear function are determined from values specified in the datasheet provided by the manufacturer [26]. The standard deviation $\sigma_{d}$ does not require any pre-processing and only uses the distance *d* in the laser depth map.

(3) $$\sigma_{d}=k_{1}\cdot d+k_{0}$$

To compute the standard deviation on edges, the training data’s edge-containing areas undergo analysis. These edges are identified utilizing the Canny edge detection algorithm on the depth map. The ensuing step involves calculating the mean standard deviation at the edges, resulting in a mean value of 0.03 m across all training images. This value is then applied to Equation (3) if the pixel location is at an edge in the validation image.

The third parameter that contributes to the total standard deviation is the angle of incidence θ. To determine it from the depth map, first, the normal map is computed by using trigonometric functions. For each pixel, the angle between the normal vector and the incidence vector is calculated. Therefore, two assumptions have been made. Since the ToF data are transformed to the RGB sensor, this minor change in perspective as well as the co-positioning of the illumination element are not taken into account. Secondly, the light direction is set to a global constant vector, which is equal to the normal of the sensor plane.

In order to determine the standard deviation based on the angle of incidence the characteristics of the sensor were investigated. For this purpose, a target plane positioned at a distance of 1 m, is captured at various angles. To achieve this, a target plane is mounted on a 3D printed platform that allows the plane to be rotated in 5° increments from 0° to 85°. The utilized material of the target plane is less reflective wood in one series, while in a separate series, the target plane made of rigid foam is captured. Therefore, an effective comparison between the two materials with different reflectivity is made.

In line with the experimental setup described by Chiabrando et al. [27], a sequence of 50 frames for each angle set is recorded. Notably, tests using a sequence of 500 frames showed no discernible difference in standard deviation as the number of frames increased.

As the angle increases, the area of the target visible to the sensor decreases while the edges of the target become more present, which can lead to inaccurate measurements. Therefore, a thin region of interest (ROI) with 5 × 20 pixels is investigated to calculate the mean standard deviation at each angle. However, since it was avoidable that even this area would be affected by higher values at the edges, the measurement at 85° is discarded.

**Figure 3:**
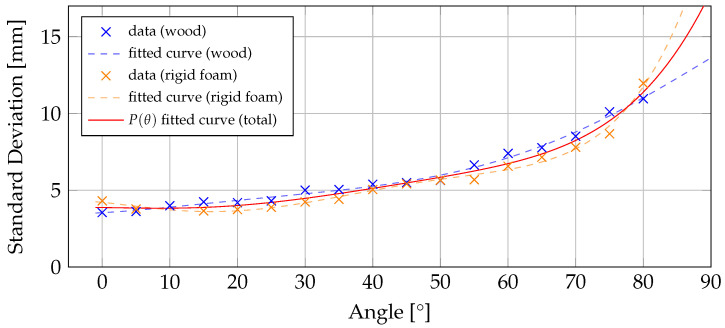
Standard Deviation as a function of the Angle of Incidence for wood (blue) and rigid foam (orange). Fitted curves are polynomials of degree 5.

Inspired by the work of Bulczack et al. [13], fitting of a polynomial is used to approximate the measured data points. For each sequence, a polynomial function of degree 5 is fitted to the measured values. The results are plotted in Figure 3. Considering that the variation among the utilized materials is relatively small in comparison to the overall range of the functions within the interval [0, 90], the final function P(θ) includes the data points from both materials.

(4) $$\sigma_{\theta }=P\left(\theta \right)-b$$

To compensate for the offset of P(θ), a bias term *b*, which $\sigma_{d}$ already incorporates, is subtracted. Combining the results from all three parameters gives the total standard deviation $\sigma_{total}$ by

(5) $$\sigma_{total}=\sqrt{\sigma_{d}^{2}+\sigma_{\theta }^{2}+\sigma_{e}^{2}}.$$

Furthermore, this leads to the resulting noise distribution

(6) $$X_{noise}∼N\left(0,\;\sigma_{total}^{2}\right),$$

where $\sigma_{total}$ is the total standard deviation matrix and $X_{noise}$ is the noise matrix around zero.

The resulting noise is finally added to the model’s predicted depth map, enabling a more realistic simulation of ToF camera behavior. It is important to note that the noise model is not used during the training phase.

## 4. Evaluation

In this evaluation, the two outcomes of the proposed method are compared with the actual ToF depth images in the evaluation set. The first outcome is the model’s prediction, denoted as PredToF. The second outcome is the model’s prediction enhanced with the noise model, referred to as SimToF.

**Figure 4:**
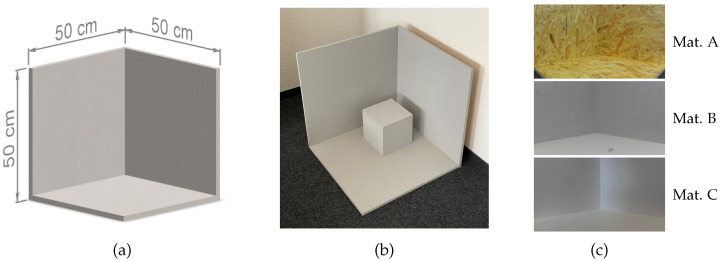
(**a**) Basic corner geometry, (**b**) photo of the corner with the shifted cube, and (**c**) RGB images of the utilized materials from the dataset.

The evaluation considers real scenes with complex geometries from the RWU3D dataset and established corner scenes that are identical to Bulczak et al. [13]. There are three different corner setups: a simple corner without any objects, a corner with an additional cube placed directly inside the corner, and the same cube shifted 10 cm away from each of the corner walls. The added cube has an edge length of 20 cm. These setups are made of grey wall paint (Material B), and white rigid foam [28] (Material C), see Figure 4. The setup with OSB panel material (Material A) is only available for the corner scene.

Cross-section plots have been recognized as a suitable visual evaluation technique in previous studies [6,13,18,21], and therefore, they are employed for the evaluation in this study. To better understand the geometry of the cross-sections, the color images are also provided. A green line is superimposed to indicate the position of the cross-section. In addition, error maps are presented to provide a global visual evaluation of the scene. These maps visually represent the differences between the actual ToF depth images and the outcomes of the methods being compared. Furthermore, to quantify the evaluation, following the approach by [13], the Mean Absolute Error (MAE), Mean Squared Error (MSE), and Root Mean Squared Error (RMSE) are utilized. Since multiple labels are available for one input, the minimum error is always reported. It is worth noting that the proposed network architecture does not generate NaN values but instead outputs small values. Consequently, any values of PredTof that are less than 0.4 m are treated as NaNs.

### 4.1. Analysis of the Training Methods

The employed variations of training methods demonstrated successful outcomes throughout the training process. The training loss consistently decreased as expected, indicating a common and desirable behavior. Furthermore, there were no signs of overfitting observed during the training. Consequently, all methods used are considered valid for evaluation purposes.

Table 1 presents the error values of the PredToF variations in the evaluation set. In contrast to the assumption that the mean loss training requires less information due to the removal of noise, our results show that this assumption is not valid. In fact, reducing the training data by using the mean loss results in a less robust model instead of the expected improvement.

Both methods that include noise to the input data could not improve the error of PredToF. Furthermore, no noticeable effects of realistic ToF noise in the prediction are observed, using this approach. Adding noise to the input data worsens the result. This indicates that the model relies on minor variations in laser depth to accurately predict the ToF behavior. By adding noise to the laser data, this crucial information is blurred, making it less effective.

On the other hand, the noise introduced in a second channel does not modify the laser data. Instead, it utilizes filters to extract features. As a result, fewer filters are available for the depth data, leading to a worse prediction.

The most successful training case is achieved by using the original, unmodified laser depth data along with the minimum loss criterion. This combination leads to the best results, with an achieved mean absolute error of 0.0533 m. Consequently, all the following analyses and evaluations are based on this particular training method.

### 4.2. Results on the Corner Scenes

SimToF achieved promising results on all corner scenes, with a mean absolute error of 0.0372 m. In contrast, the input data’s mean absolute error relative to the actual ToF on the same set is 0.1011 m. This corresponds to an improvement of more than 60%.

**Figure 5:**
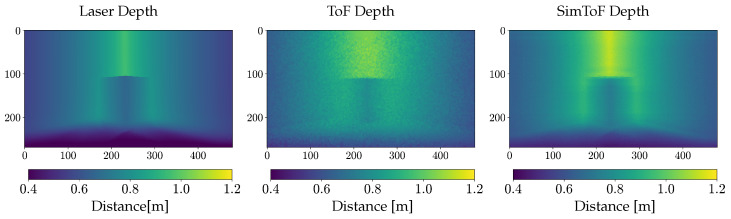
Depth image comparison between laser, actual ToF, and SimToF on the Corner Cube scene with material C.

In Figure 5, a comparison of the depth map of SimToF and the actual ToF depth map, next to the ground truth of the laser scan is shown. The visual comparison illustrates the less accurate edges and an overall higher depth of SimToF compared to the input. However, in the depth map of the ToF camera, these effects are more pronounced, and there is a notable presence of noise.

The error of SimToF with respect to the actual ToF data on the corner scenes is presented in Table 2. The results show that SimToF performs best on the corner scenes with Material C. Compared to the results of the physical simulator presented by Bulzack et al. (SimSingle, [13]) on a similar scene, the proposed method could improve the error in the Corner Cube shifted scene by 1.11 cm. However, SimSingle achieved better results on the Corner (MAE = 0.0200 m) and the Corner Cube (MAE = 0.0138 m) scene.

**Figure 6:**
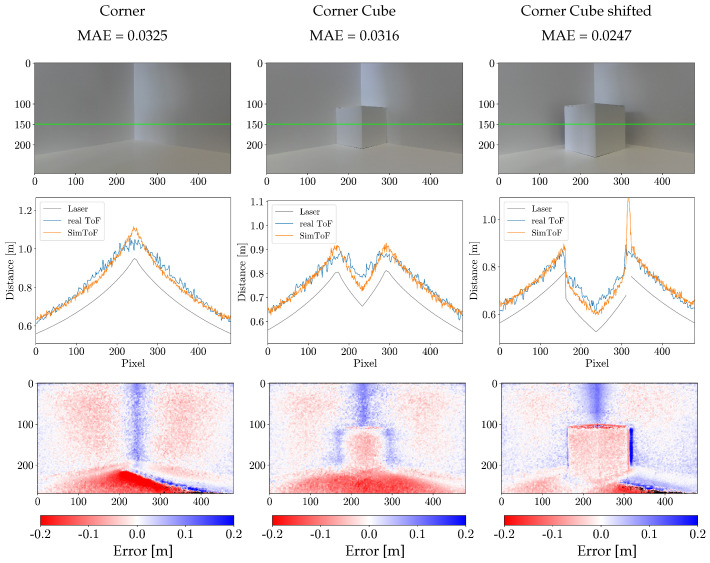
Results on the Corner Scenes with Material C: (Top row) Color images with a green horizontal line indicating the vertical position of the plots. (Mid row) Cross-section plots at row 150. (Bottom row) Error map illustrating the difference between SimToF and the actual ToF (NaN values are colored black). The columns, from left to right, represent the following: Corner, Corner Cube, and Corner Cube shifted.

Figure 6 displays the cross-section plots along with the error map showing the corner scenes. The cross-section effectively visualizes the multipath error of the actual ToF camera in the corner regions. A closer look at SimToF in this particular region shows that the MPI effects are not modeled accurately. SimToF predicts more sharp corners while the MPI is known for leading to rounded corners. In other parts of the scene, however, SimToF generates well-fitting ToF data.

The error maps reveal the main limitations of SimToF. These primarily include the vertical corner regions and areas with a flat incident angle. Both areas are prone to MPI correlated errors. Additionally, the dark blue areas indicate regions where no input data are available. To compensate for this lack of data, SimToF generates interpolation data. This characteristic can also be seen as a peak around pixel 310 in the cross-section of the Corner Cube shifted scene. In the example of the corner scenes set, the interpolation occurs on 0.5% of the data and increases the overall error by 1.08%.

### 4.3. Results on the Real Scenes

SimToF also achieved promising results on the evaluation set of real scenes, with a mean absolute error of 0.0660 m. In comparison, the mean absolute error of the input data relative to the actual ToF on the same set is 0.1694 m. This translates into an improvement of more than 60%.

**Figure 7:**
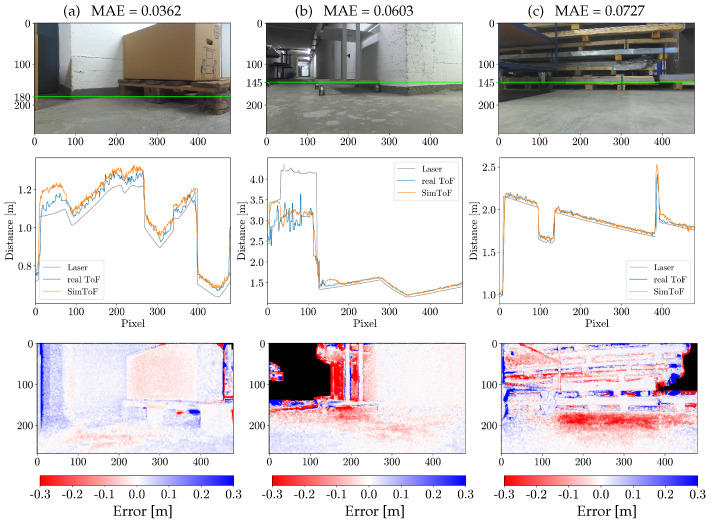
Depth image comparison of a real scene (NaN values are colored white).

Figure 7 illustrates the result of SimToF as a depth image compared to input and the real ToF camera depth image. The visualization shows that SimToF fills in the missing values in the input data, primarily found between the shelves. Additionally, SimToF produces NaN values at greater depths, similar to what the real ToF camera captures. Based on the depth images, the visual analysis shows comparable blurring results. However, the noise level of the actual ToF is noticeably higher compared to SimToF.

**Figure 8:**
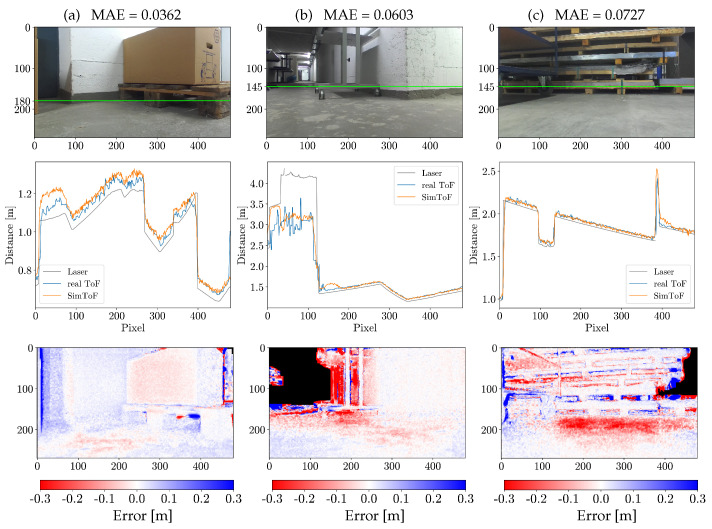
Results on samples of the real scenes evaluation set: (Top row) Color images with a green horizontal line indicating the vertical position of the plots. (Mid row) Cross-section plots. (Bottom row) Error map illustrating the difference between SimToF and the actual ToF (NaN values are colored black). The columns, from left to right, represent individual scenes labeled as (**a**–**c**).

The cross-section plots in Figure 8 provide insights into the behavior of SimToF when applied to more complex geometries. The comparison shown in Figure 8 demonstrates the accurate interpretation of the ToF camera. Furthermore, SimToF generates realistic depth data for areas where the input depth exceeds 4 m (see Figure 8b). Within this range, the actual ToF measurements fluctuate between no data and measurements that are too short. SimToF reproduces this behavior, although with higher errors compared to regions closer to the sensor.

However, there are anomalies in the prediction where higher deviations occur. In the cross-section plot of Figure 8a, between pixels 10 and 90, a recognizable higher deviation can be seen. Additionally, anomalies can be observed in the cross-section plots of Figure 8b around pixel 150 and in Figure 8c around pixel 400. Furthermore, the error map in Figure 8c reveals an error source in the floor area before the shelf. This confirms the lack of accurate MPI reproduction in SimToF, which was initially identified in the corner scenes.

In the sample scene shown in Figure 8c, SimToF shows the ability to generate realistic values for missing input data, which can be seen in the cross-section plot around pixel 390. However, similar to the corner scenes the overall error increases due to the lack of input data. Among the real scenes, 3.02% of the input data are NaN values. The higher error rates in such areas contribute to a 4.62% increase in the mean absolute error.

### 4.4. Analysis of the Noise Model

Adding noise to PredToF has a relatively small effect on the mean absolute error. The results vary due to sampling from a normal distribution. Nevertheless, the modification of SimToF allows for a better adaptation to the real ToF measurements.

**Figure 9:**
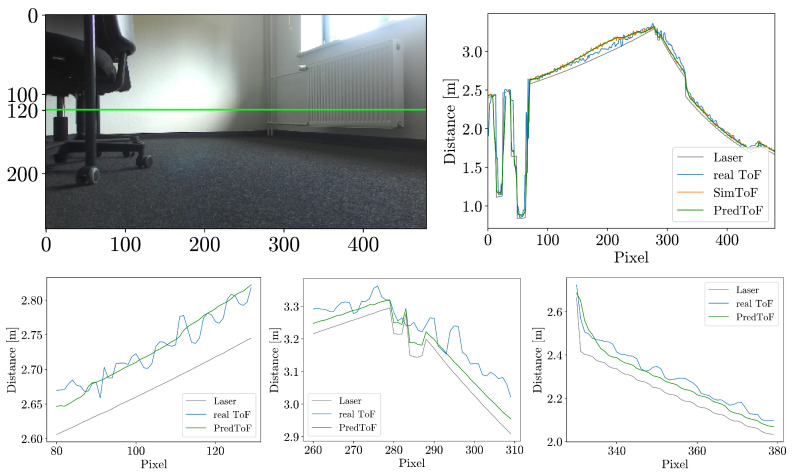

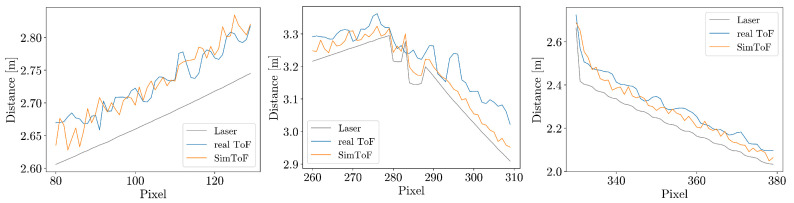
Visual comparison of PredToF (**middle** row) and SimToF (**bottom** row) on details of a horizontal cross-section plot at row 120 (**top** row, **right**). The utilized office scene from the evaluation set is displayed in color (**top** row, **left**), with a green horizontal line indicating the vertical position of all plots.Details of the Noise Model.

However, the main advantage is the introduction of more realistic noise. PredToF, in its original form, does not produce noticeable noise on flat surfaces. The generated noise by PredToF only occurs at the floors that are observed from a far distance and at a flat angle. Figure 9 provides a direct comparison of the noise characteristics. The detailed cross-section plot illustrates the significance of the noise model. SimToF aligns more accurately with the noise behavior of the actual ToF camera.

## 5. Conclusions

In summary, SimToF successfully simulates realistic ToF camera behavior and provides accurate predictions over a wide range of geometries. While there are some limitations, such as MPI correlated errors and objects in the far distance, the error maps highlight its strong performance across large image areas. Consequently, SimTof provides a reliable foundation for training neural networks. Quantitatively, this is evidenced by the fact that 84.34% of the data achieved a mean absolute error of less than 0.1 m.

One notable aspect is the ability of the network to predict the maximum measuring distance of the actual camera. Another notable feature is the generation of realistic ToF data in scenarios where no input data are available. This significantly improves the domain transfer to a ToF depth image and expands the potential applications of the model.

However, it is important to acknowledge the existing limitations at this stage. The prediction of SimToF is inaccurate, especially for objects with highly reflective materials. The evaluation revealed that none of the NaN values resulting from the filtering process of the ToF camera within its operating range are predicted. Furthermore, the noise model is restricted to one material, as it does not use material properties as input. In future work, the input data could be expanded to include RGB information to predict surface characteristics, making the noise model more adaptive and responsive to varying material properties.

## Figures and Tables

**Table 1:** Error of PredToF based on different training methods with respect to the ToF camera (values in meters). The methods are the combinations of the three distinct inputs and the two loss variants presented in Section 3.1.2.

Input		$L_{\mathrm{mean}}$	$L_{\min}$
Laser	MAE	0.0664	0.0533
	MSE	0.0285	0.0181
	RMSE	0.1349	0.1137
[Laser + Noise]	MAE	0.0977	0.0652
	MSE	0.0725	0.0311
	RMSE	0.1765	0.1381
[Laser, Noise]	MAE	0.0852	0.0631
	MSE	0.0490	0.0333
	RMSE	0.1638	0.1459

**Table 2:** Error evaluation on the corner scenes with respect to the actual ToF data (values in meters).

Material		Corner	Corner Cube	Corner Cube Shifted
A	MAE	0.0480	-	-
	MSE	0.0040	-	-
	RMSE	0.0636	-	-
B	MAE	0.0329	0.0346	0.0412
	MSE	0.0017	0.0019	0.0025
	RMSE	0.0412	0.0431	0.0503
C	MAE	0.0305	0.0310	0.0231
	MSE	0.0023	0.0021	0.0011
	RMSE	0.0478	0.0456	0.0328

## Data Availability

The data presented in this study are available on request from the corresponding author.

## Abbreviations

The following abbreviations are used in this manuscript:

ToF	Time of Flight
MPI	Multi-Path-Interference
SLAM	Simultaneous Localization and Mapping
AMCW	Amplitude Modulated Continuous-Wave
BRDF	Bidirectional Reflectance Distribution Function
RNLB	Regional Non-Local Blocks
DWT	Direct Wavelet Transform
DCR	Densely Connected Residual
SE	Squeeze-and-Excitation
PReLU	Parametric Rectified Linear Unit
MSE	Mean Squared Error
NaN	Not a Number
